# Point of Care Ultrasound (POCUS) in Bedside Diagnosis of Pyomyositis

**DOI:** 10.24908/pocus.v7i1.15288

**Published:** 2022-04-21

**Authors:** Olusegun Oduyoye, Euan Thomas

**Affiliations:** 1 Acute Medical Initial Assessment Area (AMIA), Emergency Care Centre, Aberdeen Royal Infirmary Aberdeen United Kingdom; 2 Medical Sciences & Nutrition, University of Aberdeen Medical School Aberdeen United Kingdom

**Keywords:** Point of Care Ultrasound (POCUS), Deep Vein Thrombosis (DVT), Pyomyositis, Abscess, Collection, Wells score

## Abstract

A 43 year old man with a history of IV drug use, and presenting with three days of painful and swollen left calf, was referred to exclude deep vein thrombosis (DVT). Ultrasound showed no evidence of DVT. An area of localised warm, erythematous, which was disproportionately tender, prompted a point of care ultrasound (POCUS) assessment. POCUS confirmed a hypoechoic area in the underlying tissue, likely representing a collection because of no recent trauma. It led to prompt antibiotic therapy for the treatment of his pyomyositis. The patient surgical team reviewed the patient and recommended a conservative approach with a satisfactory clinical outcome that led to a safe discharge. Overall, this case demonstrates the versatility of POCUS as an efficient diagnostic tool in the acute setting, and it also helped to differentiate cellulitis from pyomyositis.

## Introduction

Pyomyositis is an acute bacterial infection of skeletal muscle that results in localised abscess formation presenting with symptoms, including pain, swelling, erythema, and fever. It is usually associated with tropical climates; however, there has been an increasing number of cases presenting with pyomyositis in patients with a history of intravenous drug use 1, 2, 3.

The imaging modalities for the diagnosis of pyomyositis include ultrasound (US), computed tomography (CT) and magnetic resonance imaging (MRI). However, in the acute setting and after hours, point of care ultrasound (POCUS) is favoured most among Acute physicians and trainees who are skilled in using POCUS 4. It uses a portable ultrasound scanner at the bedside to help clinicians answer binary questions promptly. It is beneficial during out-of-hours and acute emergencies. POCUS is relatively inexpensive, provides real-time images at the bedside and has the added benefit of being radiation free. In the acute setting, POCUS is used for the assessment of fluids in cavities (ascites, pleural or pericardial effusions), guidance for invasive procedures (ultrasound-guided vascular access) and identifying collections 5. Kumar et al.^6^ demonstrated the effectiveness of using POCUS to diagnose and treat pyomyositis without resorting to formal imaging techniques. 

The role of POCUS in arriving at a positive early diagnosis and prompt initiation of intravenous antibiotics is a strong affirmation of one of the benefits of POCUS in an acute setting. Furthermore, the clarity of the diagnosis also facilitated early discussion with the orthopaedic team if an invasive intervention had been necessary. POCUS played a significant role in diagnosing pyomyositis during out-of-hours. The subsequent events led to a positive diagnosis (not merely a negative diagnosis) and a satisfactory patient outcome. 

## Case Report

A 43-year-old gentleman presented to the acute medical initial assessment unit with a three-day left calf swelling and pain history. He had a history of intravenous drug use, and was recently discharged from the respiratory ward two weeks prior with a right-sided empyema that required a chest drain and a course of IV Co-amoxiclav. Upon discharge, he had completed a two-week course of oral Co-amoxiclav.

The patient had a medical history of past intravenous drug use on a methadone program; previous right-sided lower limb deep vein thrombosis (DVT), chronic viral Hepatitis C, asthma and duodenitis. On admission to the acute medical initial assessment unit, he denied any recent intravenous drug use.

**Figure 1:**
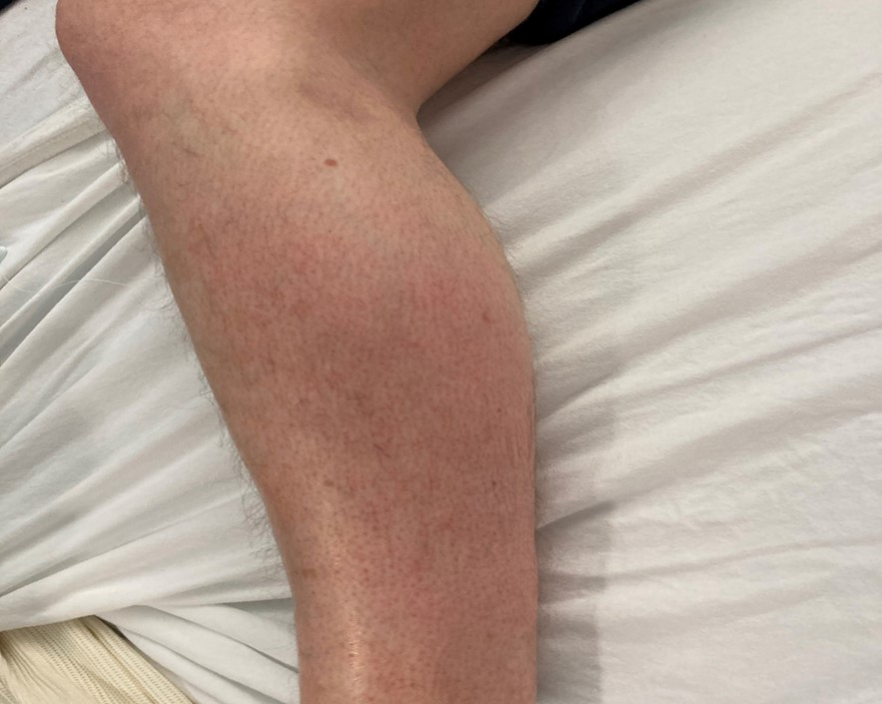
Left lateral lower limb with erythematous skin overlying gastrocnemius muscle bulk.

The patient was clinically well but complained of constant pain in the left calf on examination. The left calf was swollen and warm to the touch, with mild erythema over the lateral aspect (Figure 1). The calf was tender on palpation with no evidence of recent trauma. 

The patient had a Well's score for DVT of 4 (Calf Swelling >3 cm compared to other leg, collateral superficial veins, localised tenderness in the deep venous system and previously documented DVT). In line with NICE guidelines 7, a wells score equal or greater than two points should trigger a Doppler ultrasound scan to assess the leg veins (between the groin and the popliteal fossa) for deep vein thrombosis. He went straight for US Doppler Veins Leg Lt, which was negative for a DVT. 

During the Consultant rounds, he happened to be reviewed by the Acute Physician, who also happened to be a Focused Acute Medicine Ultrasound (FAMUS) Supervisor, who noted that there was mild erythema in the lateral aspect of the lower limb with severe tenderness on palpation. This area would not be routinely scanned when assessing the deep vein distribution during a DVT Ultrasound. 

**Figure 2:**
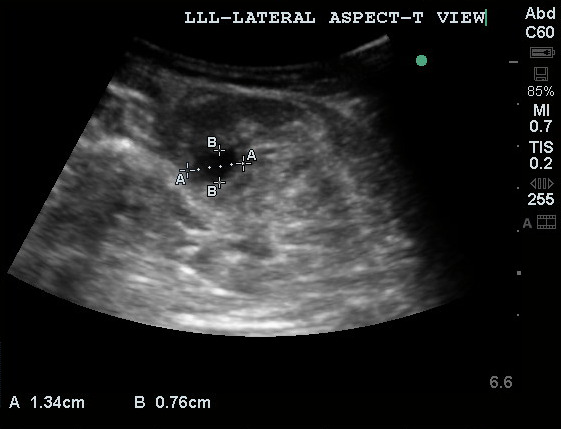
1.6×1.1cm non-compressible, non-pulsatile hypoechoic area with irregular borders within left lateral gastrocnemius muscle confirming presence of fluid.

POCUS of the lateral aspect of the patient's left leg demonstrated a 1.6x1.1cm non-compressible, non-pulsatile hypoechoic area with irregular borders likely represents a collection using bedside ultrasound (Figure 2). After that, intravenous flucloxacillin, Benzylpenicillin and Clindamycin were started to treat the collection (abscess) within the muscle as per local guidelines. 

The patient's presentation was discussed with the plastic surgery team, who noted that the current presentation is unlikely to require incision and drainage due to low inflammatory markers and the patient appearing clinically stable. They recommended continuing IV antibiotics and drainage under interventional radiology if he deteriorated.

Overnight, the lateral aspect of gastrocnemius muscle bulk (of the patient's left leg) become increasingly inflamed. The orthopaedic team reviewed the patient, who found the range of movement at the knee and ankle joints was restricted due to pain with altered sensation around the sole of the left foot. They queried a developing compartment syndrome and commenced Gentamicin, keeping the patient nil by mouth overnight for a potential surgical intervention the next day.

The following morning the patient had improved, and on assessment, there were no convincing features of necrotising fasciitis or compartment syndrome. Therefore, the patient was deemed not to require surgical intervention.

The following day the patient was well and discharged with a seven-day course of Flucloxacillin and for follow-up in the community.

## Discussion

This case demonstrates the usefulness of POCUS for quick and accurate diagnosis of soft tissue collections, especially in a busy hospital environment where the number of ultrasound scans that can be carried out each day is limited, especially after hours. POCUS can also improve patient outcomes. Patients can be scheduled for incision and drainage or guided aspiration in a timelier manner rather than prolong wait for requested scans.

A retrospective study by Trusen et al.^8^ on imaging modalities revealed that both ultrasound and MRI showed characteristic changes of pyomyositis. Ultrasound features of pyomyositis include altered echogenicity of the affected muscles and fluid collection. This was consistent with the ultrasonographic findings in our patient. However, MRI displays hyperintensity on the T2-weighted images, diffuse borders and contrast enhancement. They recommended ultrasound being the first imaging modality of choice in the extremities. 

Although Kumar et al.^6^ and Soler et al.^9^ did recommend MRI and CT scanning as the gold standard for diagnosing pyomyositis, ready access to these modalities are not always accessible in a timely fashion on a busy shift after hours. However, POCUS provides rapid diagnosis for initiation of antibiotics while MRI and CT scanning can be used for confirmation and surgical planning 6, 10. Additionally, it was noted that POCUS measurements of collections were often understated compared with MRI. Furthermore, unlike MRI, different types of fluid are difficult to distinguish from one another using ultrasound (pus, haematoma or other fluid). 

Fortunately for this patient, his clinical presentation was not as catastrophic as expected because he was on oral antibiotics from his recent discharge from the respiratory ward. This is consistent with Wang et al.^11^ work suggesting that antibiotics in the skin and soft tissue infections ameliorate the severity of inflammatory markers and clinical picture of skin and soft tissue infection. 

It is also important to note that although our patient did not require incision and drainage because of his stable clinical state, Fitch et al.^12^ recommend that most cutaneous abscesses are appropriate for incision and drainage when greater than 5 mm in diameter and accessible location. 

## Conclusion

This case report echoes the importance of maintaining a broad differential diagnosis when a positive diagnosis is not immediately in sight. It also illustrates how easy diagnosis like mild cellulitis can be differentiated from a more challenging diagnosis like pyomyositis with the aid of POCUS.

The bedside diagnosis of pyomyositis with the aid of POCUS in the hands of clinicians and trainees skilled in using POCUS is an invaluable skill. It will help prompt diagnosis of soft tissue collections (abscess) and expedite treatment in the acute setting, especially in out-of-hours.

## Statement of Consent

A written informed consent was obtained from the patient before this case was written and submitted.

## Disclosures

None.

## References

[R157090026306420] Murphy E L, Devita D, Liu H, Vittinghoff E, Leung P, Ciccarone D H, Edlin B R (2001). Risk factors for skin and soft-tissue abscesses among injection drug users: a case-control study. Clinical Infectious Diseases.

[R157090026306421] Fowler A, Mackay A (2006). Community-acquired methicillin-resistant Staphylococcus aureus pyomyositis in an intravenous drug user. Journal of medical microbiology.

[R157090026306424] Comegna L, Guidone P I, Prezioso G, Franchini S, Petrosino M I, Di Filippo P, Chiarelli F, Mohn A, Rossi N (2016). Pyomyositis is not only a tropical pathology: a case series. Journal of medical case reports.

[R157090026306416] Smallwood N, Dacshel R, Matsa A, Walden (2016). Focused Acute Medicine Ultrasound (FAMUS)- point of care ultrasound for the Acute Medical Unit.

[R157090026306423] Dietrich C F, Goudie A, Chiorean L, Cui X W, Gilja O H, Dong Y, Abramowicz J S, Vinayak S, Westerway S C, Nolsøe C P, Chou Y H (2017). Point of care ultrasound: a WFUMB position paper. Ultrasound in medicine & biology.

[R157090026306425] Kumar M P, Seif D, Perera P, Mailhot T (2014). Point-of-care ultrasound in diagnosing pyomyositis: a report of three cases. The Journal of emergency medicine.

[R157090026306417] Guidance  NICE (2020). Venous thromboembolic diseases: diagnosis, management and thrombophilia testing www.nice.org.uk/guidance/ng158. http://www.nice.org.uk/guidance/ng158.

[R157090026306414] Trusen A, Beissert M, Schultz G, Chittka B, Darge K (2003). Ultrasound and MRI features of pyomyositis in children. European radiology.

[R157090026306422] Soler R, Rodríguez E, Aguilera C, Fernández R (2000). Magnetic resonance imaging of pyomyositis in 43 cases. European journal of radiology.

[R157090026306419] Gordon B A, Martinez S, Collins A J (1995). Pyomyositis: characteristics at CT and MR imaging. Radiology.

[R157090026306418] Wang W, Chen W, Liu Y, Siemieniuk R A C, Li L, Martínez J P D, Guyatt G H, Sun X (2018). Antibiotics for uncomplicated skin abscesses: systematic review and network meta-analysis. BMJ open.

[R157090026306415] Fitch M T, Manthey D E, Mcginnis H D, Nicks B A, Pariyadath M (2007). Abscess incision and drainage. N Engl J Med.

